# Supplementary material for: Oropharyngeal microbiome profiling and its association with age and heart failure in the elderly population from the northernmost province of China
Source: Microbiol Spectr. 2024 Aug 20;12(10):e00216-24. doi: 10.1128/spectrum.00216-24 (PMC11448084; doi:10.1128/spectrum.00216-24)
Supplement: Supplemental material — Tables S1 to S5; Fig. S1 to S4. [file spectrum.00216-24-s0001.docx]

**Supplementary Information**

**Oropharyngeal microbiome profiling and its association with age and heart failure in the elderly population from the northernmost province of China**

Jian Liu^1†^, Xiao-Yu He^1†^, Ke-Laier Yang^2†^, Yue Zhao^3†^, En-Yu Dai^1^, Wen-Jia Chen^3^, Aditya Kumar Raj^1^, Di Li^1, 4, 5^, Min Zhuang^1, 4, 5^, Xin-Hua Yin^3, 6*^, Hong Ling^1, 4, 5*^

^1^Department of Microbiology, Harbin Medical University, Harbin, 150081, China.

^2^ Department of Endocrinology and Metabolism, Shenzhen University General Hospital, Shenzhen, 518055, China.

^3^Department of Cardiology, First Affiliated Hospital of Harbin Medical University, Harbin, 150001, China.

^4^ Wu Lien-Teh Institute, Harbin Medical University, Harbin, 150081, China.

^5^ Heilongjiang Provincial Key Laboratory of Infection and Immunity, Harbin, 150081, China.

^6^ Department of Cardiology, Shenzhen University General Hospital, Shenzhen, 518055, China. No.1098, Xueyuan Avenue, Nanshan District, Shenzhen, 518055, China.

* Correspondence: Hong Ling, lingh@ems.hrbmu.edu.cn; Xin-Hua Yin, xinhua_yin@163.com

**Supplementary Tables**

**Table S1.** Number of sequencing reads and depth for 16S dataset.

| **Team** | **Group (n)** | **Usable reads, mean (range)** | **Good’s coverage, mean (range)** |
| --- | --- | --- | --- |
| Age | Young (30) | 57,493 (33,121-69,796) | 0.996 (0.991-0.998) |
|  | Elderly (16) | 46,930 (29,711-63,469) | 0.995 (0.992-0.998) |
|  | Older (26) | 50,142 (41,264-62,975) | 0.996 (0.994-0.998) |
| Disease | H (32) | 47,877 (29,711-63,469) | 0.995 (0.991-0.999) |
|  | HF (32) | 46,681 (42,301-52,985) | 0.994 (0.990-0.996) |

**Table S2.** The relative abundance of six predominant phyla in age groups.

| **Young** | | | **Elderly** | | **Older** | |
| --- | --- | --- | --- | --- | --- | --- |
| **Top** | **Phylum** | **Mean** | **Phylum** | **Mean** | **Phylum** | **Mean** |
| 1 | Bacteroidetes | 0.4669 | Firmicutes | 0.4945 | Firmicutes | 0.4561 |
| 2 | Actinobacteria | 0.2068 | Bacteroidetes | 0.1520 | Bacteroidetes | 0.1596 |
| 3 | Fusobacteria | 0.1448 | Proteobacteria | 0.1426 | Saccharibacteria | 0.1496 |
| 4 | Firmicutes | 0.0858 | Saccharibacteria | 0.0921 | Proteobacteria | 0.1191 |
| 5 | Saccharibacteria | 0.0360 | Actinobacteria | 0.0618 | Actinobacteria | 0.0603 |
| 6 | Proteobacteria | 0.0263 | Fusobacteria | 0.0392 | Fusobacteria | 0.0391 |

**Table S3.** The relative abundance of high-abundant bacterial genera in age groups.

| **Young** | | | **Elderly** | | **Older** | |
| --- | --- | --- | --- | --- | --- | --- |
| **Top** | **Genus** | **Mean** | **Genus** | **Mean** | **Genus** | **Mean** |
| 1 | *unidentified_Prevotellaceae* | 0.2629 | *Streptococcus* | 0.2739 | *Streptococcus* | 0.2466 |
| 2 | *Fusobacterium* | 0.1221 | *Veillonella* | 0.1098 | *unidentified_Saccharibacteria* | 0.1226 |
| 3 | *Alloprevotella* | 0.1196 | *Neisseria* | 0.0914 | *Veillonella* | 0.1067 |
| 4 | *Rothia* | 0.0719 | *unidentified_Prevotellaceae* | 0.0818 | *unidentified_Prevotellaceae* | 0.0826 |
| 5 | *Actinomyces* | 0.0633 | *unidentified_Saccharibacteria* | 0.0708 | *Neisseria* | 0.0802 |
| 6 | *Atopobium* | 0.0262 | *Alloprevotella* | 0.0329 | *Alloprevotella* | 0.0375 |
| 7 | *Campylobacter* | 0.0258 | *Actinomyces* | 0.0280 | *Fusobacterium* | 0.0266 |
| 8 | *Leptotrichia* | 0.0221 | *Fusobacterium* | 0.0253 | *Actinomyces* | 0.0263 |
| 9 | *Porphyromonas* | 0.0216 | *Rothia* | 0.0245 | *Campylobacter* | 0.0224 |
| 10 | *Prevotella* | 0.0188 | *Oribacterium* | 0.0168 | *Rothia* | 0.0218 |
| 11 | *Lawsonella* | 0.0164 | *Gemella* | 0.0165 | *Gemella* | 0.0190 |
| 12 | *Cutibacterium* | 0.0123 | *Campylobacter* | 0.0155 | *Porphyromonas* | 0.0158 |
| 13 | *Lachnoanaerobaculum* | 0.0112 | *Haemophilus* | 0.0154 | *Prevotella* | 0.0156 |
| 14 | *unidentified_Cyanobacteria* | 0.0111 | *Prevotella* | 0.0150 | *Haemophilus* | 0.0153 |
| 15 | *Catonella* | 0.0089 | *Leptotrichia* | 0.0137 | *Oribacterium* | 0.0131 |
| 16 | *unidentified_Lachnospiraceae* | 0.0084 | *Actinobacillus* | 0.0135 | *Leptotrichia* | 0.0123 |
| 17 | *unidentified_Clostridiales* | 0.0079 | *Porphyromonas* | 0.0132 | *Megasphaera* | 0.0111 |
| 18 | *Oribacterium* | 0.0059 | *Megasphaera* | 0.0126 | *Granulicatella* | 0.0087 |
| 19 | *unidentified_Saccharibacteria* | 0.0053 | *Granulicatella* | 0.0099 | *Actinobacillus* | 0.0084 |
| 20 | *unidentified_Nitrospiraceae* | 0.0042 | *unidentified_Veillonellaceae* | 0.0071 | *unidentified_Cyanobacteria* | 0.0064 |

**Table S4.** Comparison of the relative abundance of oropharyngeal microbiota at phylum level between the H and HF groups.

| **Phylum** | **H** | **HF** | ***P*-value** |
| --- | --- | --- | --- |
| Firmicutes | 0.4868 ± 0.0156 | 0.4912 ± 0.0165 | 0.8621 |
| Saccharibacteria | 0.0822 ± 0.0058 | 0.0815 ± 0.0119 | 0.9710 |
| Bacteroidetes | 0.1597 ± 0.0079 | 0.1522 ± 0.0085 | 0.5305 |
| Proteobacteria | 0.1503 ± 0.0078 | 0.1504 ± 0.0086 | 0.9960 |
| Actinobacteria | 0.0607 ± 0.0031 | 0.0597 ± 0.0033 | 0.8402 |
| Fusobacteria | 0.0454 ± 0.0035 | 0.0451 ± 0.0031 | 0.9570 |

Data are presented as the means ± SEM. H, health group; HF, heart failure group. Differences between the two groups were analyzed by Metastats analysis.

**Table S5.** Comparison of the relative abundance of oropharyngeal microbiota at genus level between the H and HF groups.

| **Genus** | **H** | **HF** | ***P*-value** |
| --- | --- | --- | --- |
| *Streptococcus* | 0.2609 ± 0.0115 | 0.2597 ± 0.0133 | 0.9490 |
| *Veillonella* | 0.1084 ± 0.0201 | 0.1051 ± 0.0235 | 0.9430 |
| *Neisseria* | 0.0855 ± 0.0057 | 0.0831 ± 0.0064 | 0.7912 |
| *unidentified_Prevotellaceae* | 0.0865 ± 0.0044 | 0.0815 ± 0.0046 | 0.4575 |
| *unidentified_Saccharibacteria* | 0.0735 ± 0.0055 | 0.0743 ± 0.0116 | 0.9670 |
| *Alloprevotella* | 0.0324 ± 0.0025 | 0.0330 ± 0.0021 | 0.8321 |
| *Fusobacterium* | 0.0306 ± 0.0028 | 0.0301 ± 0.0023 | 0.9071 |
| *Actinomyces* | 0.0268 ± 0.0023 | 0.0252 ± 0.0015 | 0.5864 |
| *Rothia* | 0.0241 ± 0.0013 | 0.0247 ± 0.0015 | 0.7283 |
| *Gemella* | 0.0190 ± 0.0011 | 0.0242 ± 0.0022 | 0.0429 |
| *Oribacterium* | 0.0174 ± 0.0012 | 0.0186 ± 0.0010 | 0.4375 |
| *Campylobacter* | 0.0176 ± 0.0009 | 0.0181 ± 0.0009 | 0.7063 |
| *Actinobacillus* | 0.0119 ± 0.0011 | 0.0166 ± 0.0017 | 0.0329 |
| *Prevotella* | 0.0164 ± 0.0009 | 0.0158 ± 0.0009 | 0.6983 |
| *Haemophilus* | 0.0155 ± 0.0013 | 0.0152 ± 0.0010 | 0.8711 |
| *Leptotrichia* | 0.0147 ± 0.0008 | 0.0149 ± 0.0009 | 0.8541 |
| *Porphyromonas* | 0.0156 ± 0.0010 | 0.0137 ± 0.0011 | 0.2207 |
| *Megasphaera* | 0.0127 ± 0.0024 | 0.0122 ± 0.0027 | 0.9271 |

Data are presented as the means ± SEM. H, health group; HF, heart failure group. Differences between the two groups were analyzed by Metastats analysis.

**Supplementary Figures**


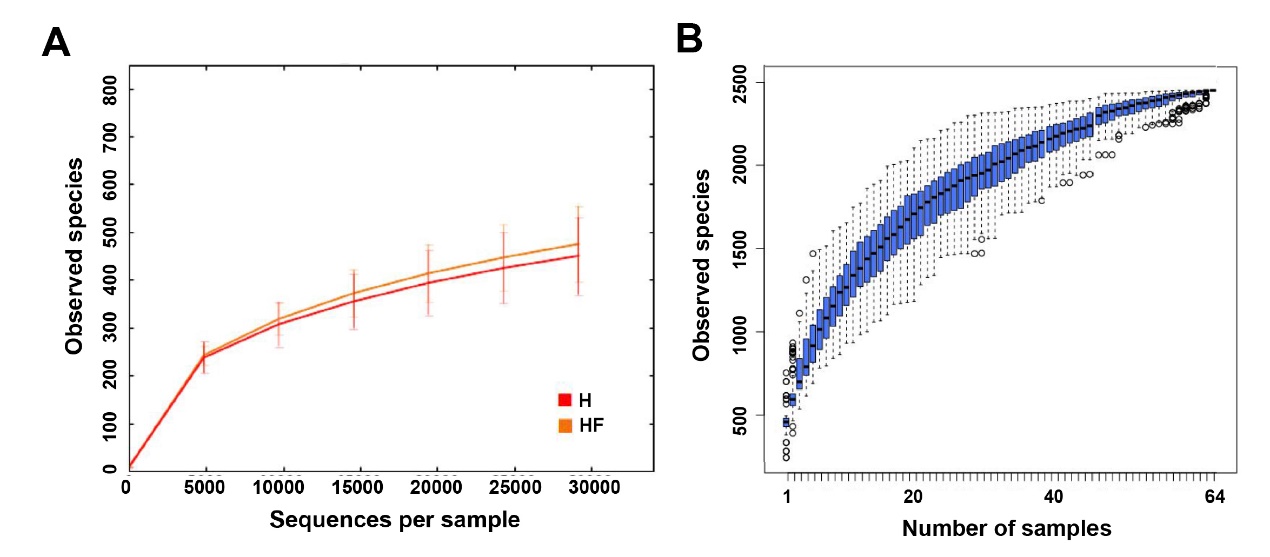


**Figure S1. Rarefaction curve and accumulation curve of oropharyngeal microbiota between the H and HF groups. (A)** Rarefaction curve. **(B)** Accumulation curve. H, health group; HF, heart failure group.

**
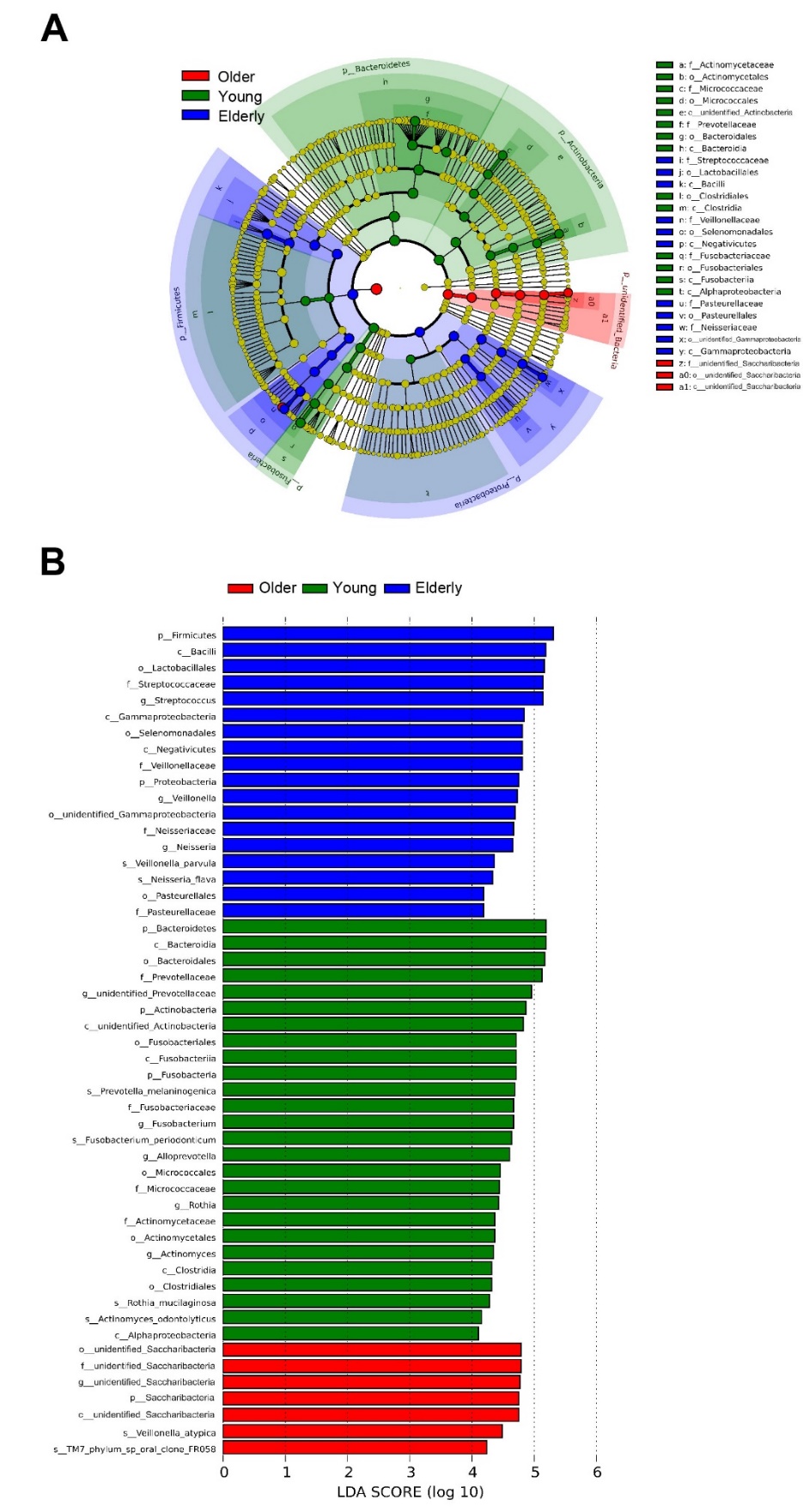
**

**Figure S2. Linear discriminant analysis effect size (LEfSe) and linear discriminant analysis (LDA) based on operational taxonomic units.** (**A**) Cladogram generated by using the LEfSe method indicating the phylogenetic distribution. (**B**) LDA scores indicated significant differences between the oropharyngeal microbiome.

**
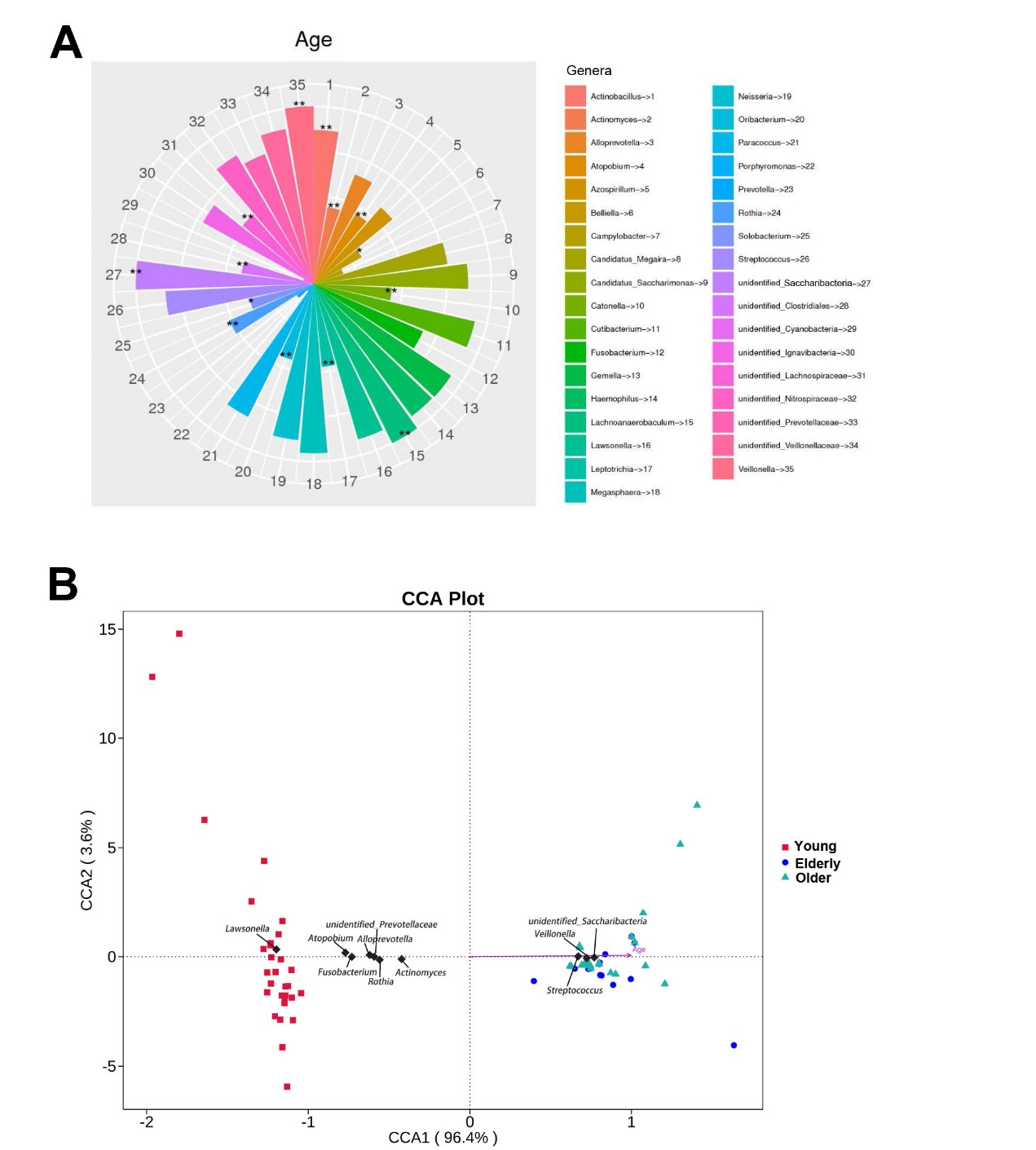
**

**Figure S3. Association between age and oropharyngeal microbiota among all healthy subjects.** (**A**) The rose chart showed the correlations between the relative abundance of bacterial genera and age. The data were following Spearman correlation analysis and indicated by * *P* < 0.05, ** *P* < 0.01. (**B**) The biplot of canonical correspondence analysis (CCA) of the microbiota composition and the age effect. Bacterial genera are shown as black diamonds, and the age effect is referred to as the arrowed line.

**
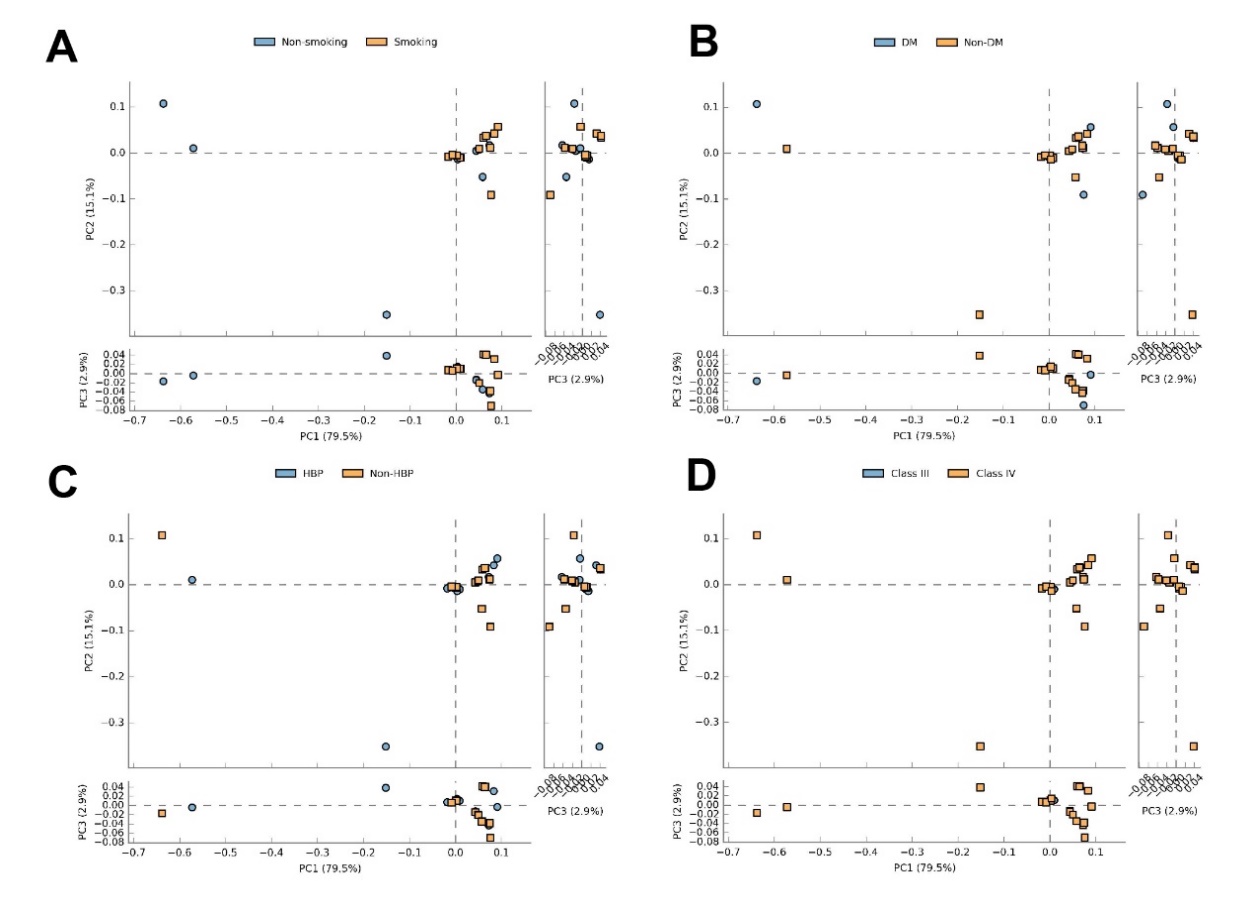
**

**Figure S4. The impact of physical statuses on the structure of oropharyngeal microbiota** **in heart failure patients.** These factors including (**A**) smoking, (**B**) diabetes mellitus, (**C**) hypertension, and (**D**) heart failure grade were analyzed by PCA.
